# *cytoHubba:* identifying hub objects and sub-networks from complex interactome

**DOI:** 10.1186/1752-0509-8-S4-S11

**Published:** 2014-12-08

**Authors:** Chia-Hao Chin, Shu-Hwa Chen, Hsin-Hung Wu, Chin-Wen Ho, Ming-Tat Ko, Chung-Yen Lin

**Affiliations:** 1 Department of Computer Science and Information Engineering, Nanhua University, No. 55, Sec. 1, Nanhua Rd., Dalin Township, Chiayi County 62249, Taiwan; 2 Institute of Information Science, Academia Sinica, No. 128 Academia Rd., Sec. 2, Taipei 115, Taiwan; 3 Division of Biostatistics and Bioinformatics, Institute of Population Health Sciences, National Health Research Institutes. No. 35 Keyan Rd. Zhunan 350, Taiwan; 4 Institute of Fisheries Science, College of Life Science, National Taiwan University, No. 1, Roosevelt Rd. Sec 4, Taipei 105, Taiwan; 5 Department of Computer Science and Information Engineering, National Central University, No.300, Jung-da Rd, Chung-li, Taoyuan 320, Taiwan; 6 Research Center of Information Technology Innovation, Academia Sinica, No. 128 Academia Rd., Sec. 2, Taipei 115, Taiwan

## Abstract

**Background:**

Network is a useful way for presenting many types of biological data including protein-protein interactions, gene regulations, cellular pathways, and signal transductions. We can measure nodes by their network features to infer their importance in the network, and it can help us identify central elements of biological networks.

**Results:**

We introduce a novel Cytoscape plugin *cytoHubba *for ranking nodes in a network by their network features. *CytoHubba *provides 11 topological analysis methods including Degree, Edge Percolated Component, Maximum Neighborhood Component, Density of Maximum Neighborhood Component, Maximal Clique Centrality and six centralities (Bottleneck, EcCentricity, Closeness, Radiality, Betweenness, and Stress) based on shortest paths. Among the eleven methods, the new proposed method, MCC, has a better performance on the precision of predicting essential proteins from the yeast PPI network.

**Conclusions:**

*CytoHubba *provide a user-friendly interface to explore important nodes in biological networks. It computes all eleven methods in one stop shopping way. Besides, researchers are able to combine *cytoHubba *with and other plugins into a novel analysis scheme. The network and sub-networks caught by this topological analysis strategy will lead to new insights on essential regulatory networks and protein drug targets for experimental biologists. According to cytoscape plugin download statistics, the accumulated number of *cytoHubba *is around 6,700 times since 2010.

## Background

Recent breakthroughs in high-throughput techniques lead experimental data deluges in genomics, proteomics, transcriptomics, metabolomics and interactomics. These data can be represented as networks, in which the nodes as surrogates for proteins, metabolites, or transcripts, are connected by edges to show the interactions, reactions, or regulations among nodes. Network analysis can help us understand the function of an individual node and the collaboration between other nodes. For example, network centralities rank nodes of a biological network according to a given importance concept, and Jeong *et al*. applied this method on a protein-protein interaction network of baker's yeast (*Saccharomyces cerevisiae*) [1]. They found that the degree of a protein correlates with the essentiality of its gene; in other word, proteins with higher degrees are more likely to be essential proteins.

Cytoscape [2] is an open platform with many plugins to expand both the visualization options and the network analysis power. Via Cytoscape, the graphical view of a network is easy accessed, and multiple layers of information including large-scale, genome-wise experiments, and protein function annotations can be granted on the interactome. Several Cytoscape plugins can score and rank the nodes by network features. For example, NetworkAnalyzer [3] and CentiScaPe [4] computes various topological network parameters for undirected and/ or directed networks. These plugins provide more centrality measures than other commonly used, but some other important features and recent developed methods are not included. Different methods focus on different topological features, or similar features with different scoring strategies. To make the network analysis easier for biologists to utilize more network features, we compose *cytoHubba *plugin to execute our newly developed algorithms and several popular algorithms. The enhanced node retrieving function in *cytoHubba *control panel helps researchers to search and explore the network and to extract user interesting subnetwork.

## Results and discussion

### The Usage of *cytoHubba*

**Figure 1:**
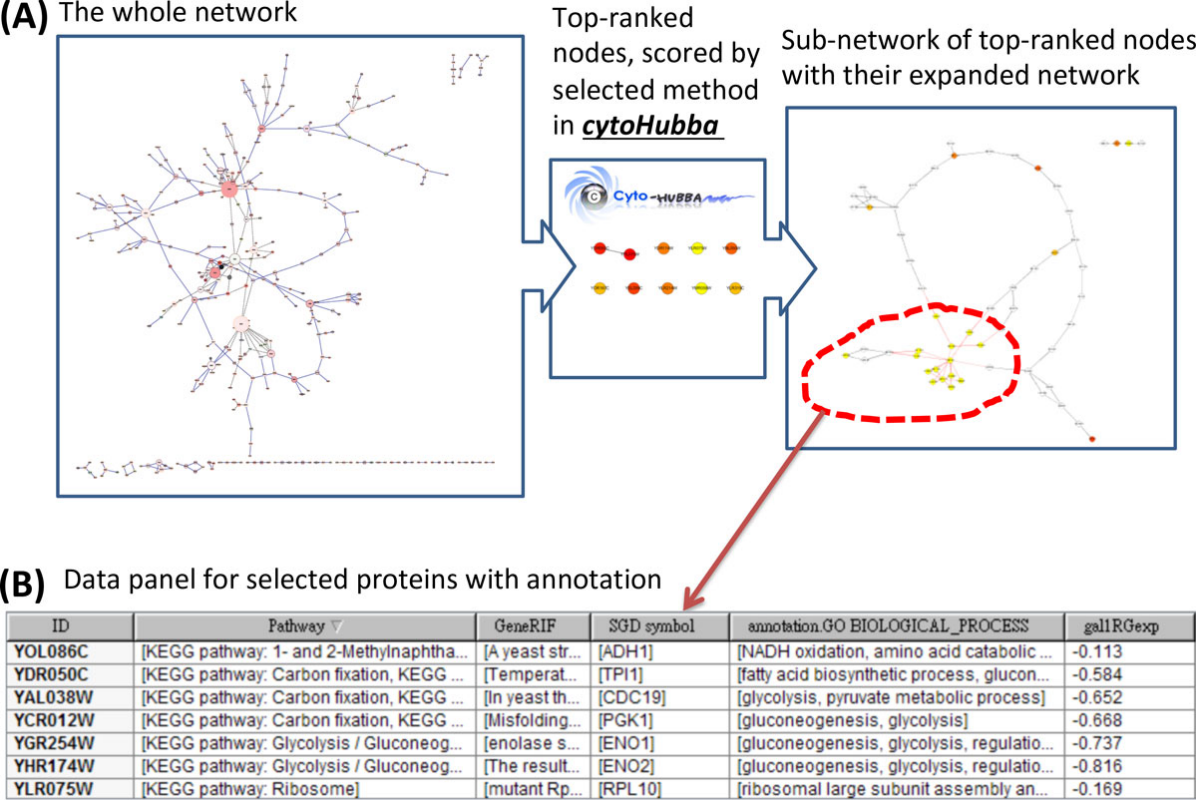
****Two screen-shots showing centrality analysis with cytoHubba****. (A) A ***cytoHubba ***analysis result using the example dataset galFiltered.cys in the Cytoscape. After the calculation (from the Cytoscape menu bar [plugins] → cytoHubba → compute hubba result), top 10 essential nodes ranked by MCC scores were selected (control panel | hubba | → select nodes→ make check on "hubba nodes"→ select a method from "choose a ranking method" and determine how many nodes are selected from "top group" → [submit])from the yeast network. (B) Node and edge annotations are accessible through Cytoscape Data Panel.

*CytoHubba *provides a simple interface to analyze a network with eleven scoring methods. First, scores from all eleven methods are granted to each node in a preloaded PPI network by executing "compute hubba result" function in the *cytoHubba *options in cytoscape menu bar [plugins]. Next, top-ranked nodes of a particular scoring method are retrieved from the *cytoHubba *tab in Cytoscape control panel, listed in the result panel, and the sub-graph of these selected nodes are shown in the main window with a color scheme from highly essential (red) to essential (green). The sub-graph of essential nodes is extendable to include nodes that directly interact with these top-ranked nodes by the option of "*check first stage node*" in control panel | hubba. Network topological features of nodes are retrievable in the data panel as options of node attributes. Tutorials and demo video are available in the website (http://hub.iis.sinica.edu.tw/cytohubba). An example of *cytoHubba *result using the Cytoscape example dataset galFiltered.cys is shown in Figure 1.

*CytoHubba *control panel is also a handy tool to retrieve subnetwork from the whole big PPI set. A list of nodes can be extracted by an ID list from the whole hubba-computed network. This manipulation can be extended to include direct linking partners (check on the option *"check first stage node"*), saved, and re-submitted to *cytoHubba *to evaluate the node essentiality on the selected sub-network. For those nodes with no direct link in between, *cytoHubba *provides a shortest path detection tool (*"display the shortest path*" on the display option). All connectible but not direct connected node pairs in a network, retrieved either by ID search or by top-ranked in topological feature score, are connected by dotted-lines with number of the smallest edge number (shortest path) to make this link. The stepping-stone nodes and edges composing the shortest path will be expanded by a mouse right-click action. Comparing with the other cytoscape plugin ShortestPath which sketches the path between two nodes [5], *cytoHubba *fetches the shortest path among a group of nodes. This abstractive view provides the distance among essential nodes.

### The performance

The studies of protein-protein interactions will be more powerful when the interactome coverage increases. However, the complexity of the network will also increase, that always hampers computation tasks. After the optimization on the programs, *cytoHubba *is able to complete all eleven analysis of a small network (e.g. 330 nodes, 360 edges), a middle size one (7,600 nodes, 20,000 edges) and a large set (11,500 nodes, 33,600 edges) in few seconds, around 30 seconds and few minutes, respectively, on a common desktop/ notebook (Cytoscape version 2.6.x / 2.7.x / 2.8.x on Window 7/8 platform; hardware spec as Intel i7, 8 GB of RAM). *CytoHubba *has been updated several times since 2009 (from v1.0 to v1.6). It is freely accessible in Cytoscape App store (http://apps.cytoscape.org/apps/cytohubba). The accumulated downloading number is around 6,500 (http://chianti.ucsd.edu/cyto_web/plugins/plugindownloadstatistics.php, statistics on May 2014). And it is used widely to analyze cancer metabolic network[6], innate immune network[7], complex biofilm communities[8] and so on.

### Validation by Predict yeast essential proteins

We use *cytoHubba *to score all proteins in the yeast protein interaction network by the eleven methods. DIP database (http://dip.doe-mbi.ucla.edu, version: 20140117) is composed of 4,908 proteins and 21,732 interactions after removing self-interactions and redundant records. The essential protein lists are collected from *Saccharomyces *Genome Deletion Project (SGDP) and *Saccharomyces *Genome Database (SGD). There are 1,122 and 1,280 proteins defined as essential proteins by SGDP and SGD respectively. We use the union set (1,297 proteins) for verifying the performance of the predictions.

The statistics of yeast PPIs are shown in Table 1. Twenty three percent (=1148/4908) of the proteins in this network are defined as essential proteins in the dataset. We call a node is high-degree if the number of its neighbors is greater than a threshold; otherwise we call it a low-degree node, where the threshold is the maximum integer such that $2\times \sum_{v\in V,Deg\left(v\right)>t}Deg\left(v\right)>\sum_{v\in V}Deg\left(v\right)$. The threshold of the PPI network (DIP 20140117) used in this paper is 21. As shown as Table 1, there are 4,396 proteins in low-degree category and 512 proteins in high-degree category, in which 908 proteins and 214 proteins are essential proteins.

**Table 1:** Statistics of Yeast PPIs used in this study (DIP database, 20140117 released set), in the aspects of degree and essentiality.

	Total	Low-degree	High-degree
The number of proteins	4908	4396	512
Essential proteins (%)	1148	908	214
	(23%)	(21%)	(42%)

Table 2 shows the performance (precision of prediction) to predict essential proteins in top × ranked node identified by each method. In most methods, the precision decreases when the selected number increase. Besides, a local-based method is better than global-based method in discovering yeast essential proteins. To further understand the preference of network feature selected by different methods, we compare the number of proteins in common in the top 100 ranked of any two scoring methods (Table 3). The top 100 ranked list of Closeness is most identical to the result of Radiality (99%), indicate that the network features detected by these two methods are very similar. MCC shares less common components to other methods (less than 30%). The top 100 ranked proteins suggested by DMNC do not appear in other methods' list except MCC (30%), means this method detect different features from the other ten methods.

**Table 2:** The performance of eleven scoring methods in predicting essential proteins, evaluated by the precision of essential proteins in the top ranked list.

Top		10	20	30	40	50	60	70	80	90	100
**Local-based Method**											
	**MCC**	0.90	0.90	0.87	0.82	0.76	0.73	0.71	0.69	0.70	0.71
	**DMNC**	0.80	0.80	0.73	0.68	0.64	0.58	0.54	0.53	0.56	0.54
	**MNC**	0.60	0.50	0.50	0.47	0.48	0.52	0.53	0.54	0.51	0.50
	**Degree**	0.70	0.55	0.47	0.42	0.46	0.47	0.49	0.47	0.46	0.44
**Global-based Method**											
	**EPC**	0.50	0.50	0.47	0.45	0.48	0.45	0.43	0.42	0.41	0.39
	**BottleNeck**	0.60	0.60	0.43	0.47	0.44	0.42	0.44	0.44	0.43	0.43
	**EcCentricity**	0.30	0.45	0.40	0.45	0.44	0.47	0.44	0.41	0.37	0.39
	**Closeness**	0.50	0.45	0.47	0.50	0.50	0.45	0.46	0.47	0.48	0.48
	**Radiality**	0.50	0.35	0.43	0.50	0.48	0.43	0.47	0.47	0.49	0.48
	**Betweenness**	0.60	0.55	0.50	0.50	0.46	0.45	0.46	0.47	0.46	0.43
	**Stress**	0.60	0.55	0.43	0.47	0.46	0.48	0.49	0.45	0.42	0.43

**Table 3:** Overlapping of the top 100 ranked list in any two scoring methods.

	Local-based				Global-based					
	**MCC**	**DMNC**	**MNC**	**Degree**	**EPC**	**BottleNeck**	**EcCentricity**	**Closeness**	**Radiality**	**Betweenness**

**DMNC**	30%	-	-	-	-	-	-	-	-	-
**MNC**	28%	0%	-	-	-	-	-	-	-	-
**Degree**	17%	0%	69%	-	-	-	-	-	-	-
**EPC**	8%	0%	60%	69%	-	-	-	-	-	-
**BottleNeck**	8%	0%	35%	53%	33%	-	-	-	-	-
**EcCentricity**	4%	0%	13%	21%	21%	23%	-	-	-	-
**Closeness**	10%	0%	63%	76%	77%	44%	29%	-	-	-
**Radiality**	10%	0%	64%	76%	77%	43%	30%	99%	-	-
**Betweenness**	14%	0%	56%	76%	54%	60%	26%	68%	67%	-
**Stress**	11%	0%	61%	88%	71%	55%	25%	77%	76%	82%

As shown as Table 1, in the yeast PPIs, 21% of proteins in low-degree category are essential proteins and 42% of proteins are essential proteins in high-degree category. Accordingly, if we pick a protein randomly from the high-degree pool, the probability that an essential protein being chosen is 0.42, and 0.21 from a low-degree protein pool respectively. Table 4 is the number of essential proteins found in the top × list with low-degree feature. For example, among the top 30 protein ranked by DMNC, 29 proteins are in the low-degree category, in which 21 out of 29 proteins are essential proteins. Methods except DMNC, MCC and EcCentricity tend to assign higher scores to a node when it owns more neighbors while almost no any low-degree proteins are found in their top × list. In other word, these methods cannot find low-degree essential proteins.

**Table 4:** The number of essential proteins found in the top × ranked list with low-degree feature.

	# of essential proteins in the low degree protein in top × ranked list | # of low degree protein in top × list									
Top	**10**	**20**	**30**	**40**	**50**	**60**	**70**	**80**	**90**	**100**

**MCC**	0 | 0	7 | 7	11 | 13	13 | 15	14 | 17	16 | 20	21 | 27	21 | 27	26 | 32	28 | 34
**DMNC**	7 | 9	15 | 19	21 | 29	26 | 38	31 | 48	33 | 56	36 | 66	40 | 76	46 | 83	50 | 93
**MNC**	0 | 0	0 | 0	0 | 0	0 | 0	0 | 0	0 | 0	0 | 0	0 | 0	0 | 0	0 | 0
**Degree**	0 | 0	0 | 0	0 | 0	0 | 0	0 | 0	0 | 0	0 | 0	0 | 0	0 | 0	0 | 0
**EPC**	0 | 0	0 | 0	0 | 0	0 | 0	0 | 0	0 | 0	0 | 0	0 | 0	0 | 0	0 | 0
**BottleNeck**	0 | 0	0 | 0	0 | 0	0 | 0	0 | 0	0 | 1	0 | 1	0 | 1	0 | 1	1 | 2
**EcCentricity**	3 | 5	3 | 8	4 | 10	6 | 12	7 | 14	9 | 18	9 | 21	9 | 24	9 | 28	12 | 31
**Closeness**	0 | 0	0 | 0	0 | 0	0 | 0	0 | 0	0 | 0	0 | 0	0 | 0	0 | 0	0 | 0
**Radiality**	0 | 0	0 | 0	0 | 0	0 | 0	0 | 0	0 | 0	0 | 0	0 | 0	0 | 0	0 | 0
**Betweenness**	0 | 0	0 | 0	0 | 0	0 | 0	0 | 0	0 | 0	0 | 0	0 | 0	0 | 0	0 | 0
**Stress**	0 | 0	0 | 0	0 | 0	0 | 0	0 | 0	0 | 0	0 | 0	0 | 0	0 | 0	0 | 0

## Conclusions

In this study, we implement our network scoring methods, MCC, MNC and DMNC, and eight other popular methods into a Cytoscape plugin, *cytoHubba*. Through the extendable, flexible and modulated properties of Cytoscape, *cytoHubba *can work together with other plugins. The computing processes had been optimized and can complete all eleven analysis on a common desktop/ notebook in a reasonable time cost. We also improve the network retrieving function in *cytoHubba *control panel. Therefore, users can utilize a PPIs network from public domain and extract sub-networks based on users' domain-knowledge.

Among the 11 methods, the newly proposed method MCC performs better than the others. MCC captures more essential proteins in the top ranked list in both high-degree and low-degree proteins. Another method, DMNC, catches different set of essential proteins suggesting it scores the network in different way. Since the biological network is heterogeneous, it is reasonable to use more than one method for catching essential proteins. We hope this handy tool can serve as good starting points to new therapies and novel insights in understanding basic mechanisms controlling normal cellular processes and disease pathologies.

## Methods

### Implementation

The *cytoHubba *plugin is implemented in Java, based on the Cytoscape API. The plugin implements eleven node ranking methods to evaluate the importance of nodes in a biological network including Degree [1], Edge Percolated Component [9], Maximum Neighborhood Component [10], Density of Maximum Neighborhood Component [10], Maximal Clique Centrality (proposed in this paper), Bottleneck [11], EcCentricity [12], Closeness [13], Radiality [14], Betweenness [15], and Stress [16]. Each method is associated with a function *F *which assigns every node *v *a numeric value *F*(*v*). We say that the ranking of a node *u *is greater than that of another node *v *if the score of *u *(i.e. *F*(*u*)) is greater that of *v *(*i.e. F*(*v*)). The 11 methods can be divided into two major categories: local and global methods. To calculate the score of a node within a network, a local rank method only considers the relationship between the node and its direct neighbors; on the other hand, the global method examines the relationship between the node and the entire network.

Text for this sub-section.

### The algorithms

#### A. Local-based Methods

Here we state notations used for describing these methods. We assume that a biological network *G *= (*V, E*) is an undirected network, where *V *is the collection of nodes within the network and *E *is the edge set. We can use another notation *G *= (*V*(*G*), *E*(*G*)) to represent a network, where *V*(*G*) is the collection of nodes in a network *G*, and *E*(*G*) is the collection of edges in a network *G*. For a set *S*, we use |*S*| to denote its cardinality (*i.e*. the number of elements in the set).

Local based method only considers the direct neighborhood of a vertex. Given a node *v, N*(*v*) denotes the collections of its neighbors. There are four local based methods shown as follows:

1. Degree method (Deg)

*Deg*(*v*)=|*N*(*v*)|.

2. Maximum Neighborhood Component (MNC)

*MNC*(*v*) = |*V*(*MC*(*v*))|, where *MC*(*v*) is a maximum connected component of the *G*[*N*(*v*)] and *G*[*N*(*v*)] is the induced subgraph of *G *by *N*(*v*).

3. Density of Maximum Neighborhood Component (DMNC)

Based on MNC, Lin *et. al*. proposed *DMNC*(*v*) = |*E*(*MC*(*v*))|/ |*V*(*MC*(*v*))|*^ε^*, where *ε *= 1.7 [10].

4. Maximal Clique Centrality (MCC)

To increase the sensitivity and specificity, we propose MCC to discover featured nodes. The intuition behind MCC is that essential proteins tend to be clustered in a yeast protein-protein interaction network [17]. Given a node *v*, the MCC of *v *is defined as$MCC\left(v\right)=\sum_{C\in S\left(v\right)}\left(\left|C\right|-1\right)!$, where *S*(*v*) is the collection of maximal cliques which contain *v*, and (|*C*|-1)! is the product of all positive integers less than |*C*|. If there is no edge between the neighbors of the node *v*, then *MCC*(*v*) is equal to its degree.

#### B. Global-based methods

In *cytoHubba *we implement six node ranking methods based on shortest paths and one method based percolated connectivity. Before we introduce the shortest based methods, let us introduce some notation. The length of a shortest path between nodes *u *and *v *is denoted as *dist*(*u, v*). Let *C*(*v*) be the component which contains node *v*. The *dist*(*u, v*) is equal to infinite if *C*(*v*) ≠ *C*(*w*), and it makes methods of this category cannot be applied to networks with disconnected components. To overcome this problem, we enhance the original methods [11-16], and the score of a node in a connected network computed by enhanced method is the same as that computed by original one.

1. Closeness (Clo)

$$Clo\left(v\right)=\underset{w\in V}{\sum }\frac{1}{dist\left(v,w\right)}$$

2. EcCentricity (EC)

$$EC\left(\text{v}\right)=\frac{\left|V\left(C\left(v\right)\right)\right|}{\left|V\right|}\times \frac{1}{\text{max}\left\{dist\left(v,w\right):w\in C\left(v\right)\right\}}$$

3. Radiality (Rad)

$Rad\left(v\right)=\frac{\left|V\left(C\left(v\right)\right)\right|}{\left|V\right|}\times \frac{\sum_{w\in C\left(v\right)}\left({\text{Δ}}_{C\left(v\right)}+1-dist\left(v,w\right)\right)}{\text{max}\left\{dist\left(v,w\right):w\in C\left(v\right)\right\}}$, where Δ*_C_*_(*v*) _is the maximum distance between any two vertices of the component *C*(*v*).

4. BottleNeck (BN)

Let *T_s _*be a shortest path tree rooted at node *s*. $BN\left(v\right)=\sum_{s\in V}p_{s}\left(v\right)$where *p_s_*(*v*) = 1 if more than |*V*(*T_s_*)|/4 paths from node *s *to other nodes in *T_s _*meet at the vertex *v*; otherwise ps(v) = 0.

5. Stress (Str)

$Str\left(v\right)=\sum_{s\neq t\neq v\in C\left(v\right)}\sigma_{st}\left(v\right)$, where σ*_st _*(*v*) is the number of shortest paths from node *s *to node *t *which use the node *v*.

6. Betweenness (BC)

$BC\left(v\right)=\sum_{s\neq t\neq v\in C\left(v\right)}\frac{\sigma_{st}\left(v\right)}{\sigma_{st}}$, where σ*_st _*is the number of shortest paths from node *s *to node *t*.

7. Edge Percolated Component (EPC)

Given a threshold (0 ≤ the threshold≤ 1), we create 1000 reduced networks by assigning a random number between 0 and 1 to every edge and remove edges if their associated random numbers are less than the threshold.

Let the *G_k _*be the reduced network generated at the *k*th time reduced process. If nodes *u *and *v *are connected in *G_k_*, set $\delta_{vt}^{k}$ to be 1; otherwise $\delta_{vt}^{k}$=0. For a node *v *in *G, EPC*(*v*) is defined as $EPC\left(v\right)=\frac{1}{\left|V\right|}\sum_{k=1}^{1000}\sum_{t\in V}\delta_{vt}^{k}$*_._*

### The demo dataset and evaluation

Database of Interacting Proteins used in this study is from DIP database ([18])(http://dip.doe-mbi.ucla.edu, version: 20140117). Essential protein lists are collected from *Saccharomyces *Genome Deletion Project (SGDP) [19] and *Saccharomyces *Genome Database (SGD) [20]. The protein ID match table from Uniprot ID to NCBI gene id is downloaded from Uniprot ftp site.

The PPI network is loaded to cytoscape and calculated by 11 methods using *cytoHubba *plugin. Precision of each method is estimated by the performance of the method to include essential proteins in the top × ranked list (x = 10, 20, 30 ..... 100) by Precision:

$$\text{Precision}=\frac{\text{the}\;\text{number}\;\text{of}\;\text{essential}\;\text{proteins}}{\text{the}\;\text{number}\;\text{of}\;\text{proteins}\;\text{in}\;\text{top}\times \text{ranked}\;\text{proteins}}$$

## Availability

*CytoHubba *is available as cytoscape plug-in and can be accessed freely at http://hub.iis.sinica.edu.tw/cytohubba/ for more detail.

## Competing interests

The authors declare that they have no competing interests.

## Authors' contributions

CHC and CYL designed the algorithm and method, and drafted the manuscript together with SHC. CHC and HHW were responsible for system implementation system and website construction. MTK and CWH participated in discussions and conceptualization as well as revising the draft. All the authors read and approved the manuscript.

## Declarations

The publication charges for this article were funded by grants MOST 100-2628-E-001 -007 -MY3 and MOST 103-2311-B-001 -033 -MY3 to CYL from the Ministry of Science and Technology (MOST), Taiwan.

This article has been published as part of *BMC Systems Biology *Volume 8 Supplement 4, 2014: Thirteenth International Conference on Bioinformatics (InCoB2014): Systems Biology. The full contents of the supplement are available online at http://www.biomedcentral.com/bmcsystbiol/supplements/8/S4.
